# Enhancing Antibody-Specific Productivity: Unraveling the Impact of XBP1s Overexpression and Glutamine Availability in SP2/0 Cells

**DOI:** 10.3390/bioengineering11030201

**Published:** 2024-02-21

**Authors:** Priscilla González-Pereira, Ryan Trinh, Alex Vasuthasawat, Angelo Bartsch-Jiménez, Constanza Nuñez-Soto, Claudia Altamirano

**Affiliations:** 1 Escuela de Ingeniería Bioquímica, Pontificia Universidad Católica de Valparaíso, Avenida Brasil 2085, Valparaíso 2340000, Chile; pgonzalezpereira@gmail.com (P.G.-P.);; 2 Department of Microbiology, Immunology, and Molecular Genetics, University of California Los Angeles, Los Angeles, CA 90095, USA; 3 Escuela Kinesiología, Facultad de Medicina, Universidad de Valparaíso, Valparaíso 2362735, Chile; angelo.bartsch@uv.cl; 4 IMPACT, Center of Interventional Medicine for Precision and Advanced Cellular Therapy, Av. Monseñor Álvaro del Portillo 12455, Las Condes, Santiago 7550000, Chile; 5 Centro Regional de Estudios en Alimentos Saludables, Av. Universidad 330, Curauma-Placilla, Valparaíso 2340000, Chile

**Keywords:** antibody, IgA, XBP1s, productivity, glutamine (Gln), SP2/0 cells, unfolded protein response, lactate, ammonia

## Abstract

Augmentation of glycoprotein synthesis requirements induces endoplasmic reticulum (ER) stress, activating the unfolded protein response (UPR) and triggering unconventional XBP1 splicing. As a result, XBP1s orchestrates the expression of essential genes to reduce stress and restore homeostasis. When this mechanism fails, chronic stress may lead to apoptosis, which is thought to be associated with exceeding a threshold in XBP1s levels. Glycoprotein assembly is also affected by glutamine (Gln) availability, limiting nucleotide sugars (NS), and preventing compliance with the increased demands. In contrast, increased Gln intake synthesizes ammonia as a by-product, potentially reaching toxic levels. IgA2m(1)-producer mouse myeloma cells (SP2/0) were used as the cellular mammalian model. We explored how IgA2m(1)-specific productivity (q_IgA2m(1)_) is affected by (i) overexpression of human XBP1s (h-XBP1s) levels and (ii) Gln availability, evaluating the kinetic behavior in batch cultures. The study revealed a two and a five-fold increase in q_IgA2m(1)_ when lower and higher levels of XBP1s were expressed, respectively. High h-XBP1s overexpression mitigated not only ammonia but also lactate accumulation. Moreover, XBP1s overexpressor showed resilience to hydrodynamic stress in serum-free environments. These findings suggest a potential application of h-XBP1s overexpression as a feasible and cost-effective strategy for bioprocess scalability.

## 1. Introduction

The most glycosylated antibody is IgA [1], and it is characterized by its ability to traverse mucosal barriers in immune defense due to its pivotal capability to form polymers (pIgA) [2]. The successful polymerization of IgA depends on the presence of the J-chain (Join chain), which is a 15 kDa polypeptide, naturally produced by cells that generate antibodies such as SP2/0 cells [3]. Specifically, its assembly is influenced by glycosylation [4], a post-transcriptional modification (PTM) occurring in the endoplasmic reticulum (ER). The ER is a highly dynamic organelle that plays an essential role in protein synthesis, folding, assembly, and PTM. It has been widely reported that the principal bottleneck for the production of secreted proteins occurs in the ER [5], where an optimal supply of machinery (foldases, chaperones, glycosyltransferases, among others) is needed [6].

Moreover, raw materials like nucleotide sugars (NS) are essential for correct assembly and glycosylation profile [7]. In the ER, the glycosylation process initiates when nucleotide sugars (NS)– uridine-diphosphate (UDP) or guanidine-diphosphate (GDP)– are sequentially attached to specific asparagine residues within the forming protein. One of the methods used to enhance NS availability includes supplementing pure NS into the culture media. However, this is costly and impractical for scaling up bioprocesses in an industrial setting. Alternatively, Gln can be added, but their metabolic pathway generates ammonia, which is thought to reach toxicity in mammalian cells after concentration levels of 2.5 mM [8] and 5 mM in human astrocytes [9], affecting glycosylation [10]. Gln, which is a nonessential amino acid due to the endogenous biosynthesis pathway, is currently considered essential in cancer cells, such as myeloma cells (SP2/0), because they consume Gln at a rate exceeding its biosynthesis [9], exhibiting a phenomenon known as Gln addiction, where the absence or deprivation of Gln triggers apoptosis [11,12]; therefore, adding Gln and evaluating ammonia synthesis is critical in mammalian cell cultures to determine if toxicity levels have been reached.

ER homeostasis can be disrupted by physiological or pathological stimuli, accumulating misfolded proteins and pushing the cell into ER stress [13]. ER addresses stress through two mechanisms: endoplasmic reticulum-associated degradation (ERAD) [14] and the unfolded protein response (UPR). The UPR relies on three ER transmembrane sensors or branches: the Activating Transcription Factor 6 (ATF6), the Protein Kinase RNA-Like ER Kinase (PERK), and the Inositol Requiring Enzyme 1 (IRE1α). The latter being the most conserved branch in yeast and mammalian –and the focus of this research– transmits signals from the ER to the cytoplasm and nucleus, regulating distinct sets of genes [15]. The IRE1α branch is a 110 kDa transmembrane protein [16] that mediates an unconventional splicing process by removing 26 intronic nucleotides from the mRNA of X-box binding protein 1 (XBP1u). Its activated form, XBP1s, subsequently relocates to the nucleus and binds to regulatory regions, orchestrating the upregulation of numerous UPR target genes [17] and various cellular processes, including metabolic homeostasis [18], fatty acid (FA) oxidation, and membrane phospholipid production [19]. It has been demonstrated that XBP1s modulates Gln metabolism [20], leading to decreased glutamine uptake by reducing transporter abundance (ASCT2, SNAT1, and SNAT2) upon XBP1 activation [21,22], inducing the transporter degradation via the ERAD pathway [23]. Even more, XBP1s plays a crucial role in protein production and immunoglobulin secretion, as its deficiency leads to a 50% reduction in secreted antibodies and an inability to effectively manage the propagation of infections caused by polyomavirus [24,25], while the knockdown of XBP1s expression in the liver demonstrated reduced cholesterol levels [26]. Furthermore, overexpression of XBP1s leads to the increase in recombinant proteins in CHO cells, NS0 cells, and HEK cells [27,28,29]. It has been suggested that overexpression of XBP1s in B-cell lines induces the expression of genes involved in enhancing the ER processing capacity, such as protein translocation into the ER, protein folding, trafficking in the secretory pathway [30], expansion of the ER, and finally increasing the overall production capacity [31]. Previous research has indicated that h-XBP1s can have both survival and apoptotic effects, migrating from adaptive to cytotoxic [17]. Despite the mechanism remaining not fully understood, it has been reported that this switch is due to a threshold in the accumulation of XBP1s protein [32]. Moreover, it has been suggested that the increase in specific productivity is directly linked to the levels of XBP1s expression in HEK 293 cells [33], and there is a positive correlation between XBP1s mRNA abundance and protein production in CHO cells [34].

This study examines the kinetic behavior of two h-XBP1s clone overexpressors characterized by presenting two levels of relative h-XBP1s protein overexpression. The investigation focuses on the effect of glutamine concentration ([Gln]), 4 mM and 8 mM, in batch cultures of SP2/0 cells producing IgA2m(1) antibody. Our objective is to establish a correlation between h-XBP1s overexpression and the specific productivity of IgA2m(1) (q_IgA2m(1)_), along with an examination of the kinetic behavior in these cultures. The results revealed a significant increase in q_IgA2m(1)_ and a reduction in ammonia accumulation, directly correlated with the level of h-XBP1s protein expression. As an additional observation, overexpressing h-XBP1s enhanced hydrodynamic resistance in stirring cultures, potentially linked to increased cholesterol fortifying cellular membranes. This study sets the groundwork for creating a highly efficient SP2/0 cell line through a combined approach of media optimization and genetic engineering. It offers a promising and economically viable platform for IgA production, shedding light on the potential of this less explored antibody in a productive context.

## 2. Results

### 2.1. Establishing a Stable Cell Line Overexpressing h-XBP1s

#### 2.1.1. Cloning Vector Construction

**Figure 1:**
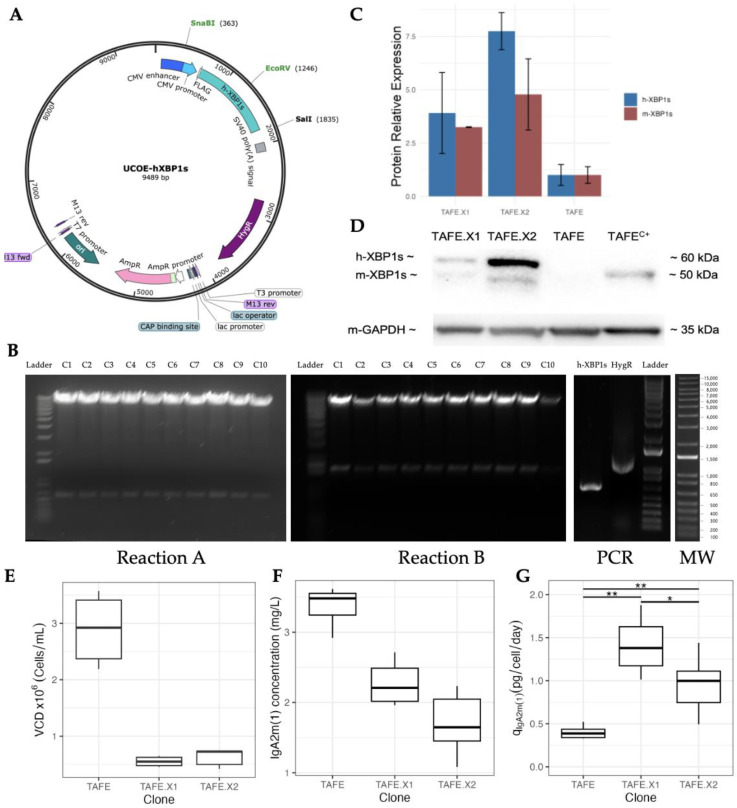
Generation of h-XBP1s overexpressor SP2/0 cell line producer of IgA2m(1). (**A**) UCOE-hXBP1s vector map, (**B**) enzymatic digestion of the UCOE-hXBP1s plasmid extracted and purified from ten *E. coli* transformed clones, Reaction A: EcoRV/SalI (8900 and 589 bp), Reaction B: SnaBI/SalI (8017 and 1472 bp), and PCR: hXBP1s (706 bp) and HygR (915 bp) amplification. C1–C10: *E. coli* clones. MW: molecular weight reference, (**C**) relative expression of XBP1s, h-XBP1s (mouse) and h-XBP1s (human) protein in the three transfectants (TAFE.X1 and TAFE.X2), (**D**) western blot of h-XBP1s protein (60 kDa), mXBP1s protein (~50 kDa), TAFE^C+^: positive control is the non-transfected clone (TAFE) treated with tunicamycin. (**E**) viable cell density, (**F**) IgA2m(1) concentration (mg/L), and (**G**) specific productivity (q_IgA2m(1)_) of the h-XBP1s protein expression (TAFE.X1 and TAFE.X2) and the parental clone (TAFE) after 72 h of culture conducted in tissue culture treated Petri dishes. Experimental values represent the means of three biological replicates. * *p*-value < 0.05, ** *p*-value < 0.01.

The precise assembly of the cloning vector, identified as UCOE-hXBP1s (Figure 1A), was validated through two enzymatic reactions: EcoRV/SalI (Reaction A: 8900 and 589 bp) and SnaBI/SalI (Reaction B: 8017 and 1472 bp). Ten transformed E. coli clones (C1 to C10) underwent plasmid extraction. All the ten extracted plasmids showed consistent and accurate enzymatic digestion fragment size, but only one was randomly selected (C1) for plasmid amplification. Subsequent confirmation of the presence of h-XBP1s (706 bp) and hygromycin resistance (915 bp) genes in the constructed plasmid was achieved, as illustrated in Figure 1B.

#### 2.1.2. Identification and Characterization of Optimal h-XBP1s Overexpressor Clones

After h-XBP1s transfection, the hygromycin-resistant genes were Ievaluated and the clones that presented increased IgA2m(1) concentration were selected (3 clones). From there, 72-h static cultures were conducted and two of those three clones, TAFE.X1 and TAFE.X2, were selected based on the relative h-XBP1s protein expression (Figure 1C,D), with TAFE.X2 displaying a higher level of the human protein. Interestingly, an increase in endogenous protein (m-XBP1s) expression was observed in the 55 kDa band, indicating crosstalk between the exogenous and endogenous proteins, possibly augmenting the role of XBP1s.

The TAFE’s static culture outperformed the transfectants in IgA2m(1) titers, reaching 3.3 mg/L compared to 2.2 and 1.8 mg/L for TAFE.X1 and TAFE.X2, respectively (Figure 1E). Simultaneously, TAFE reached a remarkable 3 × 10^6^ cells/mL, while TAFE.X1 and TAFE.X2 peaked at a modest 6 × 10^5^ cells/mL approximately (Figure 1F), clearly highlighting that the non-transfected clone prioritizes cellular growth over IgA2m(1) production. At the same time, the transfectants shifted resources to IgA2m(1) production. This conclusion is strongly supported by the specific IgA2m(1) productivity (q_IgA2m(1)_), where, in comparison, both TAFE.X1 and TAFE.X2 showed a triple and a double enhancement, respectively (Figure 1G). This compelling evidence backs the idea that XBP1 overexpression significantly boosts the antibody’s synthesis and secretion.

### 2.2. Adapting the Cells to Stirring and Low Serum Media

Following adaptation to serum-free media in tissue culture-treated Petri dishes (static conditions), the cells were inoculated into Erlenmeyer flasks and agitated at 100 rpm. After 72 h, only the h-XBP1s transfectants exhibited growth, whereas the TAFE clone showed a drastic viability drop to 20%. It has been demonstrated that the transition to serum-free conditions is challenging, and its success highly depends on the specific cell line and medium employed [35]. Further experiments with varying serum concentrations (0–10%) revealed that the minimum concentration supporting TAFE growth under agitation was 2.5%, while both transfectants thrive in a serum-free environment. This outcome suggests that the overexpression of h-XBP1s protects against the hydrodynamic stress caused by stirring.

### 2.3. Assessing Kinetics of h-XBP1s Overexpression in High Glutamine Environments

#### 2.3.1. Cell Growth and IgA2m(1) Production

**Figure 2:**
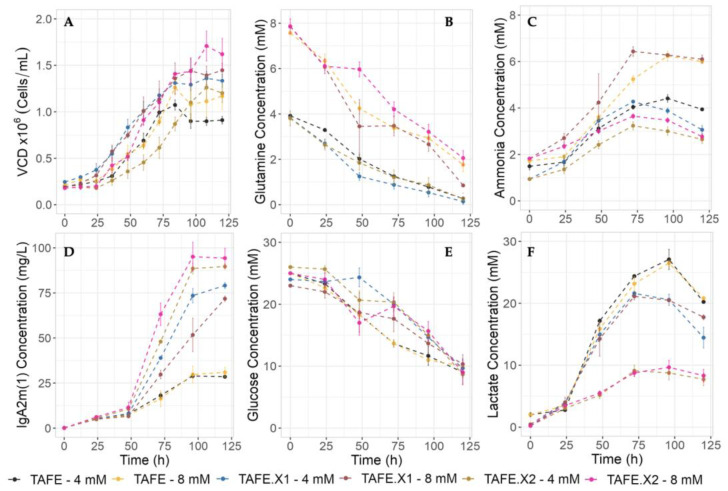
Batch culture kinetics of h-XBP1s overexpressor SP2/0 cell producer of IgA2m(1) (TAFE.X1 and TAFE.X2) and parental clone (TAFE) in agitated conditions. (**A**) Viable cell density, (**B**) glutamine consumption, (**C**) ammonia production, (**D**) IgA2m(1) production, (**E**) glucose consumption, (**F**) lactate production. Experimental values represent the means of three biological replicates. The shaded colored area around the discontinued lines represents the standard deviation.

When cultured with lower [Gln], cells entered the stationary phase at 72 h, reaching an average viable cell density (VCD) of 1.1 × 10^6^ cells/mL. In contrast, at high [Gln], the stationary phase reached 96 h, with a VCD of 1.3 × 10^6^ cells/mL (Figure 2A). The growth kinetics suggest that increasing glutamine levels could enhance culture longevity and contribute to biomass accumulation, with no discernible effect of h-XBP1s overexpression levels on growth.

During the initial 48-h phase, no signs of IgA2m(1) secretion were noted (Figure 2D). However, significant disparities in antibody accumulation patterns emerged beyond this timeframe across all cultures. h-XBP1s overexpression positively affected antibody titers, increasing by 2.5-fold for TAFE.X1 and 5.6-fold for TAFE.X2 compared to TAFE. In this context, it is evident that the levels of overexpression impact production but not growth, while glutamine impacts growth but not production.

#### 2.3.2. Glutamine Consumption and Ammonia Production

Across all culture conditions and regardless of the clone, a consistent pattern of glutamine consumption was observed. Complete depletion of glutamine occurred only under low [Gln], which indicates that it is the limiting nutrient for cell growth under this condition (Figure 2B). Simultaneously, a proportional relationship between [Gln] availability and ammonia synthesis was evident in the kinetic patterns (Figure 2C). Notably, TAFE and TAFE.X1 displayed a substantial increase in ammonia concentration in response to glutamine availability, with a 27% increase for TAFE (from 4.4 to 6 mM) and a 33% increase for TAFE.X1 (from 4.3 to 6.6 mM). In contrast, TAFE.X2 shows a modest 11% increase in ammonia production, from 3.2 to 3.6 mM, accumulating the lowest levels of ammonia compared to the other clones. These results suggest a dependent response to ammonia synthesis based on the levels of h-XBP1s protein overexpression, with higher h-XBP1s expression positively reducing the toxicity caused by ammonia.

#### 2.3.3. Glucose Consumption and Lactate Production

Since Otto Warburg discovered that mammalian cells exhibit significantly elevated glucose consumption and lactate secretion even in the presence of oxygen, cell metabolism has been a central focus for studies. Glucose consumption is similar in all cultures (Figure 2E), with remaining glucose observed even when cells have reached the stationary phase, indicating that glucose is not limiting growth in all cases. In this context, when the cells are cultured with low [Gln], this amino acid becomes limiting. However, in the presence of higher glutamine availability (8 mM), there is still an abundance of both nutrients (glucose and glutamine), indicating that the cells have additional metabolic needs that are not satisfied solely by glucose and glutamine.

Lactate metabolism is strongly influenced by the culture’s growth phase, with lactate production predominant during the exponential growth phase, and lactate re-consumption is observed when cells enter the stationary phase, as has been reported by other researchers [36] (Figure 2F). We also observed that glutamine concentration did not influence lactate production significantly. Likewise, while TAFE and TAFE.X1 consistently maintain comparable lactate production levels at the end of the culture, averaging around 27 mM and 24 mM, respectively, TAFE.X2 stands out as the clone with notably lower lactate production, only reaching 9 mM (3-fold less than TAFE).

Figure 2C,F shows a noticeable re-consumption of lactate and ammonia after reaching the stationary phase. This is consistent with the metabolic plasticity of cancerogenic cell behavior, highlighting the cells’ dynamic ability to reprogram their metabolism [37]. Cancer cells can utilize lactate as an energy and carbon source [38] and ammonia as a nitrogen source [9]. This observation provides a basis for comparison with this specific cell type.

#### 2.3.4. Impact of h-XBP1s Overexpression and Glutamine over Kinetic Parameter

The specific growth rate (μ) in the non-transfectant (TAFE) remained unaffected by [Gln], averaging 0.68 day^−1^. In contrast, the abundance of glutamine positively affected both transfectants, resulting in a similar growth rate of 0.9 day^−1^, almost 24% higher than TAFE. While at low [Gln], h-XBP1s overexpression did not significantly influence μ, as all clones exhibited similar growth rates (∼0.7 day^−1^), aligning with the findings in CHO cells overexpressing XBP1s cultured with low glutamine concentration (2 mM) [27].

**Figure 3:**
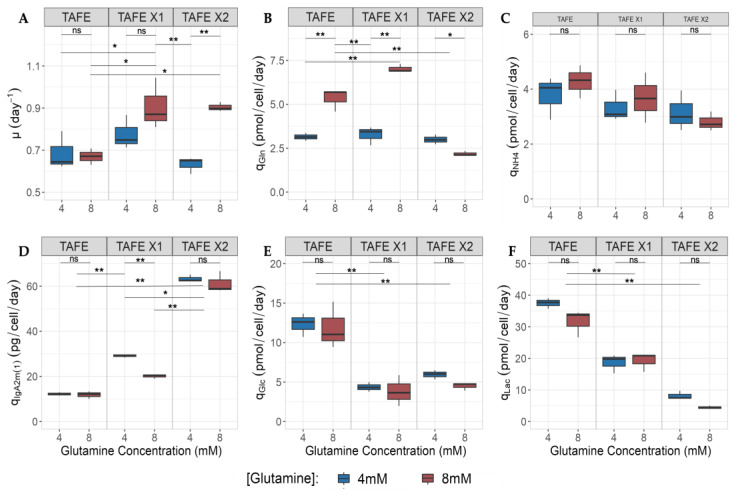
Specific consumption and production rates comparison of h-XBP1s overexpressor SP2/0 cell producer of IgA2m(1) (TAFE.X1 and TAFE.X2) and parental clone (TAFE) in batch and agitated conditions: (**A**) Specific growth rate (µ), (**B**) specific glutamine consumption rate (q_Gln_), (**C**) specific ammonia productivity (q_NH4_), (**D**) IgA2m(1) specific productivity (q_IgA2m(1)_), (**E**) specific glucose consumption rate (q_Glc_), (**F**) specific lactate productivity (q_Lac_). Experimental values represent the means of three biological replicates. * *p*-value < 0.05, ** *p*-value < 0.01.

Both transfectants substantially increased q_IgA2m(1)_ (Figure 3D). Notably, TAFE.X1 demonstrated exceptional performance under low glutamine conditions, while TAFE.X2 displayed no significant impact on glutamine abundance. In particular, TAFE.X1 achieved a q_IgA2m(1)_ of 29 pg/cell/day, nearly 2.4 times higher than TAFE’s q_IgA2m(1)_ (∼12 pg/cell/day). Meanwhile, TAFE.X2 reached 62 pg/cell/mL, representing an almost 5-fold increase compared to TAFE.

Our data suggest that the level of h-XBP1s overexpression significantly reduces the cell metabolism rate, as demonstrated in Figure 3B,C,E,F. Moreover, we have shown that glutamine availability does not impact glycolysis, exerting a null effect on q_Lac_ and q_Glc_. However, the overexpression of h-XBP1s decreased the glicolitic pathway. Where, TAFE.X1 and TAFE.X2 exhibited a 2.3-fold average decrease in q_Glc_ (∼5 pmol/cell/day) compared to TAFE (∼12 pmol/cell/day). Simultaneously, q_Lac_ decreased by almost two-fold (19 pmol/cell/day) for TAFE.X1 and 5.5-fold (6 pmol/cell/day) for TAFE.X2 compared to TAFE (35 pmol/cell/day).

However, [Gln] availability affects its own metabolism, influencing q_Gln_ and q_NH4_. In this context, when comparing the exact clone cultivated with high and low [Gln], both TAFE and TAFE.X1 behave similarly, increasing q_Gln_ with higher [Gln]. In this context, when comparing the exact clone and the two [Gln] conditions, TAFE and TAFE.X1 increase glutamine consumption by 41% (3.1 versus 5.3 pmol/cell/day) and 2.1-fold (3.3 versus 7.0 pmol/cell/day). On the other hand, TAFE.X2 reduces q_Gln_, maintaining relatively low levels (3 versus 2.2 pmol/cell/day) and decreasing its rate by 27% compared to low [Gln] cultures.

## 3. Discussion

Our findings underscore the positive impact of h-XBP1s overexpression on IgA2m(1) titers and q_IgA2m(1)_ under agitated conditions. In 96-h agitated cultures, while the parental clone achieved titers of 25 mg/L, the transfected clones—TAFE.X1 and TAFE.X2– exhibited higher titers of 75 and 100 mg/L, respectively. These titers are comparable to those reported in more advanced systems, such as bioreactors and other mammalian platforms. However, comparing IgA production with other mammalian platforms, such as CHO cells, is complex due to specific limitations. For instance, CHO cells have been shown to be suboptimal for producing polymeric IgA due to their inability to naturally produce the J-chain [3] and their incomplete assembly of IgA [39]. In contrast, SP2/0 cells have reported IgG titers of 70 mg/L after 96 h in 15-L batch cultures [40], while NS0 cells reached titers of 80 mg/L in perfusion cultures (continuous cultures with cell retention) [41]. Additionally, unpublished data from our research group indicates that NS0 cell lines produced IgG titers of 95 and 120 mg/L in batch bioreactor settings. Furthermore, it has been shown that supplementing static T-flask culture with 3 µM of methotrexate (MTX), a folic acid analogue known to enhance antibody-encoding genes, can increase titers from 50 to 300 mg/L after 6 days of cultures [42]; however effective, this supplementation may also induce mutations leading to sequence variants [43] and increase production costs. Here we developed two clones overexpressing h-XBP1s, which resulted in antibody titers comparable to those achieved in bioreactors, by only using a simple agitated platform. This indicates the potential for further titer enhancement by transitioning to a bioreactor or other culture modalities such as perfusion, continuous, or fed batch.

Even more, we showed a 2.4 and 5.5-fold increase compared to the parental clone in q_IgA2m(1)_ for TAFE.X1 and TAFE.X2, respectively, aligning with similar outcomes reported in other systems where XBP1s is overexpressed. For instance, transient overexpression of XBP1s increased protein-specific productivity by 2-fold in NS0 cells [28] and 92% in HEK cells [29]. In CHO cells, where the endogenous XBP1s factor is weakly expressed [31], overexpressing this transcription factor has proven to increase specific productivity by 5.4-fold for monoclonal antibodies [44], and by 4.5, 2.1, 5, and 2-fold for VEGF, SAMY, SEAP, and total protein, respectively [31]. Our research group has contributed to the evidence in CHO cells, showing only slight impacts of XBP1s overexpression over erythropoietin-specific productivity [27]. In B-cells, it has been shown that the mechanism behind these improvements involves the induction of genes facilitating protein translocation into the ER, protein folding, and trafficking in the secretory pathway [30], and expansion of the endoplasmic reticulum in CHO-K1-derived cell lines when XBP1s is overexpressed [31]. In the case of NS0 cells and CHO cells, XBP1s might become a determinative factor only when recombinant proteins’ accumulation exceeds the host cell’s secretory capacity, which was observed in a transient transfection system [28].

Our findings revealed a dependence on XBP1s overexpression levels for an enhanced productive setup, where the q_IgA2m(1)_ is insensitive to glutamine availability. Although the precise underlying mechanism remains elusive, research suggests that differentiated cellular responses are governed by a threshold in XBP1s protein accumulation [32]. In this context, Codamo et al. reported limited improvements in the productivity of recombinant proteins from XBP1s overexpressing CHO cells, attributed to the use of a restricted amount of XBP1s-expressing plasmid during the transfection process, leading to XBP1 dilution at an earlier stage [45]. Formas-Oliveira reported an increase in γRV (vector) specific productivity on HEK 293 cells directly related to the expression levels of h-XBP1s, reaching up to a 92% increase in specific productivity; however, lower expression of h-XBP1s has no significant effect on specific productivity [29]. The wide range of outcomes related to the expression of h-XBP1s highlights its mechanism’s cell-dependent and context-dependent nature.

ER stress has been associated with BiP/GRP78 expression in mammalian cells [34,35]. This multifunctional protein inhibits the UPR during homeostasis. Although the mechanism is not fully understood, it has been reported that BiP/GRP78 can relocate to the cell surface, especially in cancer cells [46] where significant expression facilitates the secretion of antibodies [47]. The overexpression of XBP1s results in controversial outcomes regarding BiP/GRP78 levels in different cell lines. HEK293T cells overexpressing XBP1s showed no significant differences in BiP/GRP78 mRNA or protein levels, but variations were observed, attributed to the ATF6 branch [48]. In contrast, a direct correlation was found between the levels of XBP1s mRNA overexpression and BiP/GRP78 mRNA in THP-1 cells [49]. Additionally, a significant association between BiP/GRP78 expression and endogenous XBP1s was observed in Rhabdomyosarcoma (RMS) [50]. In NIH-3T3 fibroblasts transduced with XBP1s, an increase in BiP/GRP78 expression occurred without an increase in XBP1u, attributing the elevated BiP/GRP78 levels solely to XBP1s overexpression [51].

Simultaneously, Crespo et al. observed a counterintuitive effect when glutamine levels (10 mM) increased in epithelial cells treated with an ER stress-inducing compound, such as tunicamycin (TUNI). Their experimental approach was to expose the cells to TUNI for 12 h, demonstrating partial attenuation of the upregulation of the BiP/GRP78 protein [52]. The limited 12-h TUN treatment is a point of comparison with our cell cultures overproducing lower levels of h-XBP1s (TAFE.X1) that demonstrated adverse effects of increasing glutamine levels, potentially linked to a reduced expression of BiP/GRP78, which might result in the limited secretion of IgA2m(1). Jiang et al. observed that in 24-h cultures of epithelial cells with Tunicamycin (TUN) supplemented with 7 mM glutamine as well as in a glutamine-free medium, higher levels of BiP/GRP78 were achieved in the presence of elevated glutamine concentration [46]. This prolonged cellular stress mirrors our highly overexpressor clone, TAFE.X2, which shows increased qIgA2m(1). However, since these authors did not evaluate low glutamine concentrations, it remains uncertain whether the effects were due to elevated glutamine availability or the mere presence of glutamine. Our data suggest high h-XBP1s overexpression boosts secretory capacity irrespective of glutamine concentration; however, this needs to be further addressed in future research to delve deeper into the underlying mechanism and comprehensively understand the observed effects.

It has been proposed that glutamine concentration stimulates q_Gln_ rate and q_NH4_ [53], which is valid for the TAFE.X1, which expresses lower levels of h-XBP1s and at higher [Gln] also increases q_Gln_. Likewise, it has also been demonstrated that upregulation and induction of XBP1s downregulated the abundance of glutamine carriers (SNAT1, SNAT2, and ASCT2), limiting the glutamine influx in T-cells [22,54], via degradation of the carriers via ERAD [24]. This phenomenon was observed in our higher h-XBP1s overexpressor clone (TAFE.X2), which maintained basal q_Gln_ regardless [Gln]. Our study also revealed that h-XBP1s overexpression, particularly at higher levels, decreased q_Glc_, q_Lac_, and overall lactate and ammonia accumulation. However, the reported outcomes in the literature are diverse. It has been shown that XBP1s overexpression leads to an increase in lactate production (1.6-fold) [27], suggesting that XBP1 enhances glycolysis upregulation in immune cells [41], promoting lactate production [55]; on the other hand, the knockout of XBP1s does not impact the expression of genes related to glycolysis, glucose uptake, or lactate production [56], and XBP1s silencing results in decreased lactate production [57]. Here, we confirmed that the XBP1s role is context-dependent and varies across cell types and specific conditions [58], such as the level of h-XBP1s expression and [Gln] in the context of oncogenic cells like SP2/0, which seems to be that their metabolism is continuously adapting for survival.

Our findings revealed a notable decrease in glycolysis pathway activity upon h-XBP1s overexpression (by reducing lactate titer, q_Glc_ and q_Lac_), while glutamine titer and q_Glc_ levels remain unaffected by h-XBP1s abundance. Notably, q_Lac_ demonstrates a direct correlation with heightened levels of h-XBP1s. This observation is consistent with prior research indicating that XBP1s can influence glucose homeostasis independently of the unfolded protein response (UPR) mechanism [59]. Additionally, it has been proposed that XBP1s directly stimulates the hexosamine biosynthesis pathway (HBP), where glucose is utilized for antibody glycosylation. Consequently, this activation leads to the downregulation of several rate-limiting enzymes in the glycolysis pathway [60] and the suppression of Glucose Transporter 1 (GLUT1) expression via RNA polymerase II recruitment [61]. Consequently, this cascade results in decreased ATP abundance. The decrease in ATP abundance subsequently enhances pyruvate production to fuel oxidative phosphorylation (OXPHOS) [62], potentially leading to reduced lactate synthesis.

As a baseline, our parental clone (TAFE) stood out as a highly productive cell line, yielding IgA2m(1) titers nearly 2000 times higher than other SP2/0 cells producing IgG, typically around 1.5 μg/L in static conditions [63]. Even more, when TAFE was cultured under stirring conditions, it showed a nine-fold increase in IgA2m(1) compared to static conditions. However, the most striking outcome was observed in the transfectants, which demonstrated a remarkable leap in IgA2m(1) titers by a 20- and 89-fold increase for TAFE.X1 and TAFE.X2, respectively, compared to their static counterparts. While prior reports have acknowledged the significance of agitation in boosting productivity, the enhancements observed in this research are even more significant. Foster et al. showed that cultivating SP2/0 cells under agitation resulted in a mere three-fold increase in TNFα specific productivity compared to cultures in static vessels [64]. The increased productivity under stirring compared to static conditions can be explained by the negative impact of the low oxygen availability in the environment occurring in static conditions. It is widely acknowledged that oncogenic cells, including SP2/0 cells, demonstrate an increased demand for oxygen during their development, and the tumor microenvironment is recognized for its characteristic hypoxic conditions [65]. In this context, as cells approach a high VCD in the early stationary phase, they may confront an oxygen-limited environment. Notably, the TAFE clone has shown an enhanced capacity to adapt to this environment, enabling it to achieve higher VCDs than cells overexpressing h-XBP1s.

Additionally, it was observed that in static cultures, the h-XBP1s overexpressor reached a low VCD, almost 6-fold less than the non-transfectant (parental clone), which the hypoxic environment can also influence. Although the mechanism is not fully understood, it has been suggested that hypoxia can induce endoplasmic reticulum (ER) stress due to the accumulation of reactive oxygen species (ROS) [66]. Under this condition, XBP1s inhibits the expression of antioxidant genes, such as catalase, superoxide dismutase 1 (SOD1), and cofactors like thioredoxin 1 (TRX1) mRNA, thus reducing the cell’s ability to maintain balanced ROS levels [67]. Excessive or chronic ROS production can result in oxidative stress, causing an imbalance between ROS generation and the cell’s capacity to detoxify and repair ROS-induced damage. Therefore, the combined effect of oxygen limitation and h-XBP1s overexpression could lead to an enhanced accumulation of ROS, impacting cell proliferation.

We demonstrated that h-XBP1s overexpression exerts a protective effect on hydrodynamic stress (induced by stirring). In this context, research confirms the dependency of cells like SP2/0 and other myeloma cells, such as NS0 cells, on external lipid supplementation (serum or lipoprotein additives) [68]. This reliance is associated with membrane lipid composition, in particular cholesterol abundance. Studies have demonstrated that shear stress reduces cholesterol in cell membranes, mitigated by cholesterol supplementation in culture media [69]. Interestingly, our findings indicate an improved overall culture performance (increased growth and q_IgA2m(1)_) when the cells overexpress XBP1s in an agitated setup than in static conditions. In this context, XBP1s have been linked to the direct upregulation of several lipogenic genes, and recent studies have shown that in cancer cells, such as myeloma cells, XBP1s promote the synthesis and secretion of cholesterol [70] by redistributing the metabolic glucose flux to lipids biosynthesis [71]. Emerging evidence suggests that overexpression of XBP1s in tumor cells stimulates cholesterol biosynthesis by directly binding to the promoters of genes essential to this process. For instance, it has been demonstrated that overexpressed XBP1s binds to the promoter of HMGCR, a key enzyme in the cholesterol synthesis pathway [70]. We hypothesize that this outcome is related to robustness in the membrane composition of the transfectants due to an increase in cholesterol synthesis mediated by XBP1s.

In summary, we have developed a clone capable of producing antibody concentrations comparable to those achieved in bioreactors under agitated (Erlenmeyer-flask) culture conditions correlated to the overexpression of h-XBP1s. We observed an increase in secretory capacity, possibly due to the upregulation of BiP/GRP78 and its relocation to the cell membrane and increase in ER processing machinery. Additionally, we achieved a reduction in lactate production, possibly due to limitations in glucose transporters and a shift in glycolytic flux towards lipid (cholesterol) formation, ATP through OXPHOS, and glycosylation. We also observed a decrease in ammonia synthesis, possibly due to a decrease in glutamine transporters. Furthermore, we obtained clones that were more tolerant to hydrodynamic stress, a crucial aspect for scaling up production processes.

## 4. Limitations

We evaluated the effects of hXBP1s overexpression in only two single cell clones, where cell productivity could be explained as a direct consequence of our cell engineering approach, clonal variance, or both. For example, we observed variation in h-XBP1 protein expression levels between the TAFE.X1 and TAFE.X2 clones, which can be attributed to several factors inherent in the cloning process, such as transfection efficiency, genomic integration sites of the transfected plasmid, and the presence of clonal diversity within the SP2/0 cell line itself. The post-transcriptional and post-translational regulatory mechanisms may also contribute to the observed differences in protein expression levels between the clones. This underscores the inherent complexities in industrial mammalian cell line development, where epigenetic modifications during single-cell cloning can lead to phenotypic divergences from the original cell pool [72]. To reduce the effect of clonal variance on our outcomes, we took measures to mitigate the impact of epigenetic silencing by constructing an expression vector with a constant, permissive open state in chromatin structure, ensuring consistent, stable, and high-level gene expression regardless of chromosomal integration sites.

A second potential source of variability stemmed from passaging and age-related issues. To address this issue, we implemented a rigorous cloning storage and cellular bank protocol to minimize clonal variations and maintain experimental reproducibility.

The last important step to reduce the impact of clonal variance on our results lay in the statistical methods. We opted for robust statistical methods, specifically employing robust analysis of variance (Robust ANOVA). This choice was driven by the recognition that conventional statistical assumptions, such as homogeneity of variances (homosce-dasticity), are difficult to confirm from both experimental and statistical perspectives. By aiming to mitigate the impact of potential violations of these assumptions, our approach not only minimizes the risk of statistical errors but also enhances the reliability of our results, ensuring a more robust and confident interpretation of our findings.

A secondary limitation involves the use of SP2/0 cells, a murine myeloma cell line. Despite the industry’s predominant focus on CHO cells, the utilization of SP2/0 cells expressing IgA2m(1), a heavily glycosylated protein, represents a challenging expression candidate that offers a unique and insightful case study. Notably, our results demonstrated increased cell productivity comparable to that observed in other mammalian cell lines, such as CHO and NS0 cells, suggesting that the effects of hXBP1s overexpression may be replicable across various mammalian cell lines, lending a degree of generalizability to our findings. However, our methodology and scope of work does not allow us to make inference to other cell lines, and future research should encompass a broader spectrum of clones, to assess potential phenotype variations.

Despite these limitations, the use of SP2/0 cells overexpressing h-XBP1s proves valuable as it allows us to explore the ramifications of secretion pathways and cell productivity in a novel context. This offers the potential to unveil new avenues for optimizing biopharmaceutical manufacturing.

## 5. Materials and Methods

### 5.1. Cell Culture and Media

In the Morrison Lab at UCLA, IgA2m(1)-producing SP2/0 cell lines were developed using a previously documented methodology [73,74]. This cell line, named TAFE, was used as the host in this study and was the parental clone for the following experiments. The cells were grown in a 60 × 15 mm Tissue Culture Petri Dish (Corning^TM^, Gilbert, AZ, USA) with 5 mL of IMDM media (Iscove Modified Dulbecco’s Medium, Gibco #124400, Carlsbad, CA, USA) supplemented with 5% *v*/*v* calf serum (R&D System #S11950, Minneapolis, MN, USA) and incubated in a 5% CO_2_ environment at 37 °C. IMDM is commercially formulated with 4 mM of glutamine. Therefore, considering the choice of this medium and the desire to conduct experiments with 8 mM of glutamine, the need for glutamine supplementation took precedence. The decision was influenced by avoiding introducing additional variability to the results that might arise from using another more stable source of the amino acid (alanyl-glutamine). To assess glutamine stability, cultures with and without cells were examined, revealing that the observed decrease in glutamine concentration was solely attributable to cell growth rather than instability. Even more, to ensure consistency and address potential instability associated with storage, glutamine supplementation was carried out immediately before use.

The cells were routinary amplified when cells reached nearly 90% confluence (approx. 72 h) and passage into a 100 × 20 mm tissue culture Petri dish with 15 mL of serum-supplemented media to an initial concentration of 2.5 × 10^5^ cell/m. Cell counting was performed using the Trypan Blue exclusion assay [75], and the viable cell density (VCD) was calculated.

### 5.2. Construction of the h-XBP1s Cloning Vector

The h-XBP1s cloning expression vector was constructed utilizing the UCOE^®^ Mu-H (Sigma Aldrich #504866, San Diego, CA, USA) and the CMV-hXBP1s (Addgene #63680, Watertown, MA, USA). The parental plasmid possessed the UCOE^®^ (Ubiquitous Chromatin Opening Element) technology to boost gene expression in stably transfected mammalian cells. It maintains a constant, permissive open state in chromatin structure, preventing epigenetic silencing, as it has been reported to render CHO cell lines resistant to gene-silencing effects [76]. This ensures consistent, stable, and high-level gene expression regardless of the chromosomal integration site.

The interest fragments were obtained through restriction digestion using the enzymes SalI (Thermo Fisher ER0641, Freemont, CA, USA) and SnaBI (NEB R0130S, Ipswich, MA, USA). The enzymes were applied sequentially in two steps due to distinct optimal reaction conditions with a purification step in between utilizing the Qiaquick^®^ Gel Extraction Kit (QIAGEN #28706, Germantown, MD, USA) following the manufacturer’s instructions. Calf phosphatase treatment (Invitrogen #18009-019, Carlsbad, CA, USA) was applied to UCOE^®^ Mu-H digestion. The reaction was visualized in an agarose gel electrophoresis, and the interest fragments were excised with a scalpel. Purification was repeated, and a T4 DNA ligase performed the fragment ligation step (Invitrogen^TM^ #15224017). The new plasmid, named UCOE-hXBP1s, contained the h-XBP1s gene, the hygromycin resistance (HygR) gene (mammalian cell selection), the ampicillin resistance gene (bacterial selection), among all the required elements.

### 5.3. E. coli Transformation, Purification, and Characterization of the UCOE-hXBP1s Cloning Vector

The UCOE-hXBP1s cloning vector underwent amplification by heat shock transformation of competent *E. coli* HB101 cells and cultured in selective media (LB broth supplemented with 200 mg/L ampicillin). Cells were harvested at 6000× *g* for 15 min, and plasmid extraction and purification were performed using the QIAGEN Plasmid Maxi Kit (Qiagen #12162), following the manufacturer’s protocol. The UCOE-hXBP1s concentration was quantified at 260 and 280 nm wavelengths using a microplate in a BioTek Synergy^TM^ HT spectrophotometer.

The integrity of the UCOE-hXBP1s construct was confirmed through two enzymatic restriction reactions, A: EcoRV (NEB #R3195S) and SalI (Thermo Fisher #ER0641), and B: SnaBI and SalI, and the presence of h-XBP1s and HygR genes was assessed by polymerase chain reaction (PCR) using the Platinum^TM^ Taq polymerase (Invitrogen^TM^ #10966018). Primers were designed using the Primer3Plus Software, version: 3.3.0 (https://www.primer3plus.com/index.html, accessed on 23 May 2023) and analyzed using the IDT OligoAnalyzer tool (h-XBP1s forward: 5′-CTGGAACAGCAAGTGGTAGAT-3″ and Reverse: 5′-TAGGCAGGAAGATGGCTT-TG-3″ and HygR gene primers Forward: 5′-TTCAGCTTCGATGTAGGAGGGC-3″ and Reverse: 5′-TTCCTTTGCCCTCGGACGAGTGC-3″. Amplification conditions were set at 94 °C for 2 min, 30 cycles at 95 °C for 1 min, 50 °C for 1 min, and 72 °C for 1 min, followed by a final extension at 72 °C for 10 min and kept at 4 °C.

### 5.4. Stable Lipotransfection of IgA2m(1) Producer SP2/0 Cells with the UCOE-hXBP1s Plasmid

Following the manufacturer’s instructions, the UCOE-hXBP1s vector was introduced into the host cell through stable transfection using DMRIE-C Transfection Reagent (Invitrogen^TM^ #10459-014). The cells were then resuspended in serum-supplemented media and distributed across five 96-well plates (Day -2). Two days later (Day 0), hygromycin-supplemented media was added. Two days later (Day 2), the media in each well was replaced, and colonies were allowed to form (approximately 14 days). Colonies were individually amplified upon isolation and amplification; the colonies were immediately frozen and stored in liquid nitrogen to create a master bank. Subsequently, cells from the master bank were expanded to produce a working bank of 14 new criovials. One criovial from the working bank was defrosted for each new set of experiments. Upon exhaustion of the working bank, a new master bank criovial was expanded, reiterating the process. This cryovial preservation and banking strategy aimed to minimize passaging-induced variability, ensuring consistency in experimental conditions.

A prior dose-response curve of hygromycin (antibiotic killing curve) was performed to determine the lowest toxic concentration of the antibiotic that killed the host cells (non-transfected).

Finally, our primary objective was to identify highly productive clones and assess the impact of culture conditions and overexpression. Therefore, the selection of the overexpressor clones was based on, first the level of overexpression of h-XBP1s and the increase IgA2m(1) production (concentration).

### 5.5. h-XBP1s Relative Expression: Western Blot

h-XBP1s relative expression was normalized against the housekeeping GAPDH protein. The samples were prepared as follows. In total, 3 × 10^6^ cells underwent lysis using RIPA Lysis Buffer (Thermo Fisher #8900). The resultant protein lysate was quantified by BCA Protein Assay Kit (Thermo Scientific #23225, Carlsbad, CA, USA) following manufacturer’s protocol, and 30 μg of protein was subjected to SDS-PAGE gel electrophoresis on NuPAGE^TM^ 12% Bis-Tris Gel (Invitrogen #NP034BOX) for approximately 40 min at 90 volts. The protein bands were then transferred to a PVDF membrane (30 volts for 1 h), and the membrane was excised following the molecular weight. The membrane fragments were then incubated with blocking solution (5% non-fat milk and 2% tween 20) and incubated under agitation for 2 h at room temperature, after three-times washing with wash solution (1% tween 20 disputed in PBS 1×). The fragments were incubated accordingly with primary antibodies, anti-h-XBP1s (Cell Signaling #12782, Danvers, MA, USA) and anti-GAPDH (Sigma #G9545), diluted in the blocking solution 1:1000 and 1:10,000, respectively, and incubated in agitation overnight at 4 °C. After 3 washes, a secondary antibody, anti-IgG-HRP (Cell Signaling #7074), was added and incubated under agitation for 2 h at room temperature, and after that another 3-times wash was performed. Finally, the membranes were incubated with a Chemiluminescent Substrate (Super Signal^TM^ West Pico Plus, Thermo Scientific #1863097), prepared as manufacturer’s instructions. The bands were visualized and captured using a digital chemiluminescent detector (Azure Biosystem C280, Dublin, CA, USA). Normalization was performed using Image J 1.53t software (version 1.53) to ensure accurate sample comparisons. We utilized a pre-stained molecular weight marker to confirm the complete transfer of proteins to the PVDF membrane. The presence of bands solely on the PVDF membrane indicated successful transfer. The Western blot accuracy was ensured by including a negative control lysate (parental clone lysate) and an endogenous positive control (TAFE cells treated with tunicamycin).

### 5.6. IgA2m(1) Quantification: ELISA

The concentration of IgA was measured using an Enzyme-Linked Immunosorbent Assay (ELISA), in clear flat-bottom immuno 96-well plates (Thermo Fisher #3355). The plates were coated with anti-human IgA antibody (capture antibody), prepared by dissolving 5 μg/mL of IgA-alpha-chain specific from rabbit (Sigma #I9899) in sterile PBS 1×. 50 μL of this solution was added to each well and incubated at 4 °C overnight, followed by three washes with PBS 1×. Then, 50 μL of 3% *w*/*v* BSA in PBS 1x was added to each well, and the plates were incubated for at least 30 min or stored at 4 °C for up to 2 months. After washing the plates with PBS 1×, 50 μL of properly diluted supernatant of cultures (in PBS 1×) was added to each well in triplicate and incubated overnight at 4 °C. The sensitivity range was from 0 to 0.1 μg/mL, and a standard curve was conducted on every plate. The next day, the plates were washed with PBS 1×, and 50 μL of alkaline phosphatase-labeled anti-human kappa light chain antibody (Sigma #A3813). Diluted 1:10,000, was added to each well. The plates were then incubated for 2 h at room temperature. After another three washes with PBS 1×, 50 μL of freshly prepared phosphatase substrate (Sigma #50942) was added. The substrate was diluted in diethanolamine buffer (pH 9.8), prepared by mixing diethanolamine (Sigma-Aldrich #D8885), 1 M MgCl_2_, and distilled water, with pH adjustments using HCl 10 N. The plates were read at 410 nm before absorption reached saturation, typically within an hour at room temperature, with a 30-min incubation being sufficient for this purpose. Consistency in incubation time was crucial for reproducibility across experiments.

### 5.7. Cell Culture Platform for Agitation and Low Serum

Since these clones have never been adapted to agitation nor serum-free conditions, the cells were adapted to serum-free media by gradually reducing the serum concentration at each passage, starting from 10% and progressively decreasing to 5%, 2.5%, 1.25%, 0.06%, 0.03%, and eventually to 0%. Once the cells adapted to serum-free media, they were inoculated into Erlenmeyer flasks under agitation at 100 rpm. The cultures were closely monitored until the cells decreased their viability to 70%.

### 5.8. Batch Culture of h-XBP1s Overexpression Cells

Cultures were conducted in 250 mL vented PETG Erlenmeyer flasks (Cell Treat #229825, Pepperell, MA, USA), each containing 50 mL of media. Two concentrations of glutamine –4 mM and 8 mM– were evaluated in the study. For the 8 mM cultures, the media was supplemented with L-glutamine (Gibco^TM^ #25030081), whereas supplementation was unnecessary for the 4 mM cultures as the media already contained this concentration. Samples were collected, and cell counting using the trypan blue exclusion technique, IgA2m(1) quantification and metabolic analysis were performed. Every culture was performed in triplicate.

### 5.9. Metabolic Analysis of Lactate, Ammonia, and Glucose

Glucose (Glc), lactate (Lac), and ammonia (NH_4_) was quantified through the Biosystems Analyzer Y15 (Kit #12532, BioSystems SA, Barcelona, Spain), while glutamine (Gln) concentration was assessed with a YSI 2700 Biochemistry Analyzer from Yellow Springs Inc. (Yellow Springs, OH, USA).

### 5.10. Specific Rate Calculation

The determination of the cell-specific growth rate (μ), IgA2m(1)-specific productivity (q_IgA2m(1)_), and rates of metabolite consumption/production (q_Glc_, q_Gln_, q_Lac_, and q_NH4_) was carried out using well-established methodologies outlined in previous studies [77].

### 5.11. Statistical Analysis

A Robust ANOVA was performed to test for statistical differences between cell cultures. This choice was made due to several unmet critical assumptions required for a traditional ANOVA test. These assumptions included normal distribution of data, homogeneity of data dispersion (heteroscedasticity), as well as a sufficiently large sample size [78]. Applying a Robust ANOVA offered a more reliable and accurate approach to statistical inference, especially considering the limited triplicates conducted for each culture (n = 3). Moreover, Robust ANOVA offers the possibility of testing more than one main effect and their interactions in one single step, which further reduces statistical error when compared with non-parametric tests, such as Kruskal–Wallis. As such, we tested the three main effects and interactions (Glucose × Temperature × Clone type) in a single Robust ANOVA, instead of three separate Kruskal–Wallis tests.

In cases where the null hypothesis was rejected, a post hoc analysis using the Conover method was conducted to further investigate the observed differences. The significance level (Type I error) for all tests performed was set at 5% (*p*-value < 0.05). All statistical analyses were conducted using R and RStudio software (version 2021.09.1 Build 372).

## 6. Conclusions

We established a novel SP2/0 mammalian cell platform for enhanced q_IgA2m(1)_, by combining two strategies, media design and genetic manipulation. Here, high h-XBP1s overexpression exhibited reduced metabolic rates, effectively mitigating toxicity associated with lactate and ammonia accumulation, and enhanced resistance to hydrodynamic stress, eliminating serum dependence for growth. This innovative platform demonstrates a cost-effective IgA production, serving as a baseline for potential applications in bioprocessing setups. However, inferring these outcomes to other cell lines and other recombinant proteins is not warranted and further research is needed.

## Data Availability

The data will be available upon request to the main author.
